# Drug-Integrating Amphiphilic Nano-Assemblies: 3. PEG-PPS/Palmitate Nanomicelles for Sustained and Localized Delivery of Dexamethasone in Cell and Tissue Transplantations

**DOI:** 10.3390/pharmaceutics17101337

**Published:** 2025-10-16

**Authors:** Giulio Palummieri, Saeida Saadat, Sung-Ting Chuang, Peter Buchwald, Diana Velluto

**Affiliations:** 1 Diabetes Research Institute, School of Medicine, University of Miami, Miami, FL 33136, USA; gxp929@med.miami.edu (G.P.); saeidasaadat@miami.edu (S.S.); sxc2158@med.miami.edu (S.-T.C.); pbuchwald@med.miami.edu (P.B.); 2 Department of Molecular and Cellular Pharmacology, University of Miami, Miami, FL 33136, USA

**Keywords:** polymeric nanoparticles, glucocorticoids, drug delivery, inflammation, immunotherapies, allogeneic transplantation

## Abstract

**Background:** Glucocorticoids are an important class of therapeutics used in a variety of applications, including allotransplantations. Dexamethasone (Dexa) is well-known for its strong anti-inflammatory, immunosuppressive, and anticancer properties. However, its clinical use is often limited by its poor water solubility, poor pharmacokinetics, and high likelihood of systemic side effects. **Methods**: To address the issues, we tested a combined strategy where our original Drug-Integrating Amphiphilic Nano-Assemblies (DIANAs), a class of self-assembling polymeric nanoparticles designed for controlled drug release, were used to solubilize and deliver dexamethasone palmitate (DexP), a hydrophobic prodrug of dexamethasone. **Results**: The palmitate chains of the prodrug can form strong van der Waals interactions with the hydrophobic moieties of the PEG-PPS block copolymer used here. In water, this resulted in the self-assembling of stable dexamethasone palmitate–PEG–PPS nanomicelles, termed DexP-nMICs, with a 25 nm average diameter that slowly released Dexa over more than two weeks. **Conclusions**: Here we demonstrated that DexP-nMICs can carry elevated amounts of Dexa—increasing its solubility in water—prolong circulation in its pharmacologically active form in vivo and provide passive targeting to inflammation sites. The anti-inflammatory efficacy of DexP-nMICs was first confirmed in vitro on stimulated macrophages, demonstrating a significant reduction in cytokine secretion. An allogeneic mouse skin transplant model, used to assess the therapeutic potential of DexP-nMICs in vivo, confirmed its ability to provide graft-targeted delivery and prolong graft survival as compared to the unformulated parent drug. Therefore, DexP-nMICs are a promising candidate for sustained and localized use of anti-inflammatory drugs in cell and tissue transplantations.

## 1. Introduction

Drugs, either natural or synthetic, have always been designed and used to treat or cure a disease or medical condition and improve the quality of a patient’s life. Unfortunately, in certain clinical situations, many therapeutic agents currently in use can cause undesired effects that limit their pharmacological benefits, with glucocorticoids being a notorious example. Other well-known examples include anticancer drugs and chronic systemic immunosuppressive therapies for patients who have received cell or tissue transplantation.

Due to our interest in beta-cell replacement therapies in patients with Type 1 Diabetes (T1D), our focus is on improving the needed life-long immunosuppressive treatments [1]. T1D is a chronic autoimmune disease caused by the selective destruction of insulin-producing pancreatic beta cells by autoreactive T cells [2,3,4]. This leads to an inability of the body to regulate blood glucose homeostasis, resulting in chronic hyperglycemia, the need for exogenous insulin administration, and a high risk of severe complications, including kidney failure, coronary heart disease, diabetic retinopathy, neuropathy, and cardiovascular disease [5,6]. Transplantation of pancreatic islets represents a promising alternative to exogenous insulin therapy and has the potential to cure T1D by restoring beta cell function and insulin production [7,8,9]. This method improves glucose counter-regulation, enhances hypoglycemia symptom recognition, and normalizes glycemia, reducing dependence on exogenous insulin [10]. The pioneering work of Lacy et al. [11,12] and the development of a semi-automated human pancreatic islet isolation process by Ricordi et al. [13] were instrumental in transitioning islet transplantation from an experimental procedure to a viable clinical therapy [9,14]. Since 2000, several hundred people have received islet transplants with encouraging results [14,15].

Despite significant progress [16], the widespread application of islet transplantation remains limited by two major challenges: the scarcity of donor islets and the need for chronic systemic immunosuppression to prevent graft rejection [17]. While the first is being addressed by developing stem-cell-derived islets [18,19,20], systemic immunosuppression is still required to ensure long-term graft survival [21]. Indeed, from 1999 to 2010, the long-term insulin-independence rate of clinical islet transplantation has been steadily improving, but only if combined with potent systemic immunotherapy [22], which is associated with severe side effects, including renal toxicity, cardiovascular morbidity, and increased susceptibility to infections and malignancies [23,24]. Furthermore, the majority of immunosuppressant drugs, such as cyclosporine A (CsA) [25], sirolimus (rapamycin, Rapa) [26,27], and tacrolimus (FK506) [25], as well as anti-inflammatory agents, such as the glucocorticoid dexamethasone (Dexa), also suffer from poor water solubility, poor stability, unfavorable pharmacokinetics, and unavoidable systemic side effects, making their use challenging [28,29].

A successful localized immunosuppression and anti-inflammatory (LISAI) therapy could enhance islet engraftment and survival while eliminating or minimizing systemic toxicity [30,31]. In recent years, nanoparticle-based therapeutic approaches have demonstrated significant potential for addressing the limitations of conventional administration and improving delivery of drugs [32,33]. The opportunity to achieve tunable targeting properties, high drug-carrying capacity, favorable size distribution, and ease of surface functionalization makes nanoparticles a favored approach for LISAI regimens.

Towards such goals, the work conducted by our group in the field of controlled polymer synthesis over the past decade has allowed us to achieve varying and stable architectures at the nanoscale, enabling fine-tuning of nanoparticle size, morphology, surface chemistry, and drug-loading capacity to optimize pharmacokinetics and biodistribution [30,34,35,36,37,38].

Using a living polymerization technique, our group has previously engineered amphiphilic block copolymers with controllable molecular weights, hydrophilic fractions, and polydispersity that are able to self-assemble and form nanoparticles in a single step without the need for toxic cross-linkers. Furthermore, by simply modifying the block copolymer length and ratio during the polymerization, nanoparticles, termed DIANAs (Drug-Integrating Amphiphilic Nano-Assemblies), can be obtained with different sizes, shapes, biodistribution behavior, and drug loading efficiency [30,36]. Therefore, for an LISAI regimen, DIANAs were designed to encapsulate hydrophobic drugs such as immunosuppressant agents, dramatically increasing their water solubility and stability and mediating their localized and sustained effect [30,36]. We have shown that CsA-loaded DIANAs effectively inhibited the proliferation and activation of cytotoxic T cells in vitro. In mice, subcutaneous injections of CsA-loaded DIANAs enabled prolonged CsA release, reducing alloantigen-induced immune responses in draining lymph nodes at lower doses, with less-frequent administration compared to unformulated CsA [30]. Biodistribution studies with fluorescently labeled DIANAs demonstrated their ability to accumulate at inflammation sites via passive targeting after systemic administration [30,36]. Therefore, DIANA nanoparticles represent a versatile platform for localized immunosuppression with potential applications in allogeneic cell transplantation.

**Figure 1:**
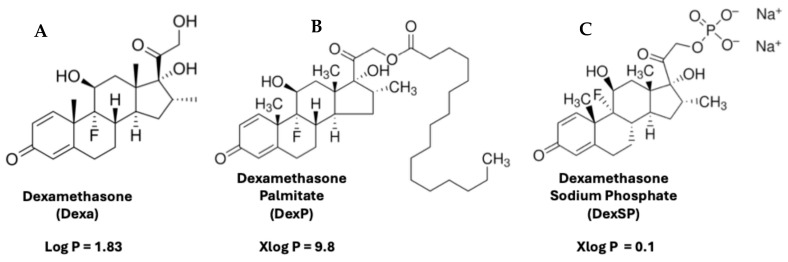
Chemical structures of Dexamethasone (**A**), Dexamethasone Palmitate (**B**) and Dexamethasone Sodium Phosphate (**C**), drugs used in this study, and their log *p* values (log *p* values shown are experimental for dexamethasone and calculated as XlogP for dexamethasone palmitate and for dexamethasone phosphate, as obtained from PubChem).

Besides immunosuppressives, another essential class of drugs that can benefit from localized delivery is anti-inflammatory agents. The first common response to the introduction of foreign cells and tissue is, indeed, the inflammatory reaction at a graft site. This reaction is triggered by the body’s immune system attempting to recognize and remove the graft, and leads to inflammation at the site of implantation, causing early rejection. Therefore, inflammation must be prevented or reduced, and glucocorticoids (GCs) can be particularly useful as they also have immunosuppressive effects. Among GCs, Dexa is widely used for its anti-inflammatory, immunosuppressive, and anticancer properties. The immunomodulatory effects of glucocorticoids like Dexa stem from their ability to regulate B- and T-cell activity. Dexa has gained considerable attention due to its affordability, availability in multiple formulations, well-characterized mechanism of action, and diverse administration routes [39,40]. Despite its potent pharmacological efficacy, the clinical use of Dexa is often limited by its poor water solubility and its poor pharmacokinetics (short half-life, poor absorption, and poor distribution through the body). An improvement in the poor and unfavorable pharmacokinetics of dexamethasone can be achieved with the use of its hydrophobic prodrug, dexamethasone palmitate (DexP, Figure 1A), which is obtained by modifying the parent drug [41] with the addition of a long fatty acid chain via an ester bond that is easily cleaved intracellularly [42,43].

This prodrug has already been shown to prolong the half-life of the active compound Dexa from minutes–hours to days–weeks [42]. However, since the presence of a C_16_ chain confers the molecule a very high hydrophobicity, as revealed by its considerably increased log octanol–water partition coefficient (computed Xlog P of 9.8 versus the experimental log P of 1.83 for the active molecule Dexa; Figure 1), administering DexP remains a challenge. On the other hand, this more hydrophobic cargo loads better and releases more slowly from the DIANA nanoparticles. Therefore, we prepared DIANA nanoparticles, in particular nanomicelles (nMICs) made from self-assembling amphiphilic block copolymer PEG_44_–PPS_20_ [30], to load, stabilize, slowly release, and target to inflammation sites clinically relevant amounts of DexP, ultimately improving the pharmacological properties of Dexa and reducing its side effects in vivo. Because the DexP-loaded nanomicelles (DexP-nMICs) are only around 25 nm in diameter, they can quickly extravasate and be absorbed by the immune cells infiltrating an inflammation site [30], thus releasing their anti-inflammatory payload at their desired site of action. The strategy of local release of glucocorticoids could also overcome another problem caused by their systemic administration. Dexa and other corticosteroids cause impairment in insulin-stimulated glucose uptake and an increase in glucose production in the liver. This can lead to impaired glucose tolerance and insulin resistance, potentially developing into steroid-induced diabetes [44]. Their prolonged administration is known to produce whole-body insulin resistance and exacerbate diabetes [45]. The systemic administration of corticosteroids in the peri-transplant period has been associated with the development of islet graft dysfunction [46], and steroid-free regimens are now the norm in clinical settings for islet transplantation [47]. An LISAI regimen for Dexa can avoid or reduce this metabolic interference that is particularly dangerous for patients with T1D.

Here, we report the outcomes obtained with our new DexP-nMIC-nanoparticle-based treatment in vitro in a macrophage cytokine secretion model as well as in vivo in a full-thickness murine skin transplantation model, which is a well-established in vivo model for studying alloimmune responses and graft rejection. The goal was to prove the efficacy and safety of our nanoparticle-based LISAI regimen for future translation into clinical use in islet transplantation for patients with T1D.

## 2. Materials and Methods

### 2.1. Materials

All chemical reagents and HPLC-grade solvents were purchased from Sigma-Aldrich (St. Louis, MO, USA) and from VWR (Radnor, PA, USA). Dexamethasone palmitate was obtained from Sigma-Aldrich; dexamethasone and dexamethasone sodium phosphate were obtained from MedKoo Bioscience (Durham, NC, USA). The cell culture products were provided by Thermo Fisher Scientific (Waltham, MA, USA). Cytokine ELISA kits were obtained from R&D Systems (Minneapolis, MN, USA), the dexamethasone forensic ELISA kit from Neogen (Lansing, MI, USA), and the human insulin ELISA kit from Mercodia (Winston Salem, NC, USA).

### 2.2. Synthesis of Polymeric Amphiphilic Diblock Copolymers

**Figure 2:**
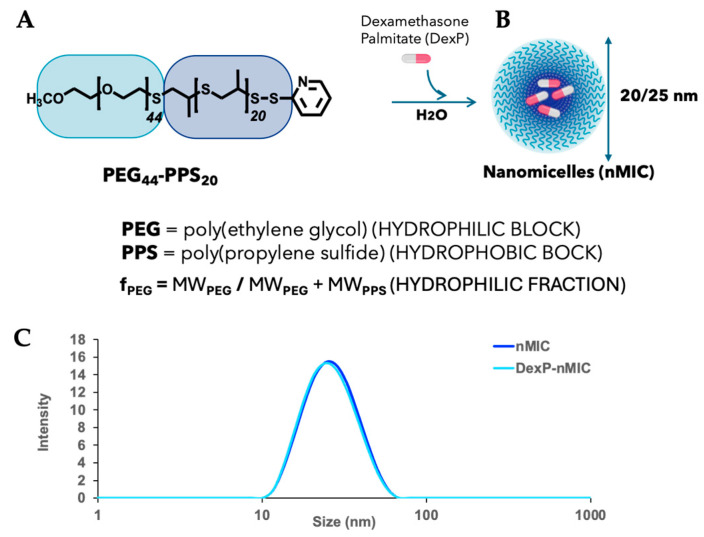
Self-assembling of PEG_44_–PPS_20_ into drug-loading nMICs. Schematic of the chemical composition of poly(ethylene-glycol)–poly(propylene sulfide) (PEG_44_–PPS_20_) diblock copolymers with hydrophilic fraction of 0.57 (**A**) and self-assembling in water to form nanomicelles (nMICs) loaded with the drug cargo (DexP) (**B**). (**C**) Example of size distribution for the nMICs (blue line) and the DexP-nMICs (light blue line) analyzed by dynamic light scattering with a Malvern zeta-sizer.

Poly(ethylene glycol)-poly(propylene sulfide) copolymer of desired length (PEG_44_–PPS_20_) (Figure 2A) was synthesized via anionic-ring-opening polymerization of (PS) from a thiolate PEG macroinitiator as previously reported [30]. Briefly, a linear monomethoxy-poly(ethylene glycol) (mPEG–OH, MW 2 kDa) was modified to obtain a thiol-protected group on the -OH end of the chain (m-PEG-thioacetate); then, the thiol was activated in the presence of 20 equivalents of monomer propylene sulfide (PS) to initiate the anionic-ring-opening polymerization. After 90 min, all the monomer reacted, and the poly–PS chain terminus was reversibly capped by disulfide exchange with 2,2′-dithiodipyridine to provide PEG_44_–PPS_20_-s-s-Py (Figure 2A). For simplicity, the polymer composition will be indicated as PEG_44_–PPS_20_ in the rest of the manuscript. Therefore, this block copolymer can be further functionalized, if necessary, by a disulfide exchange reaction between any thiolate molecule and the thio-pyridine at the end of the PPS block [48]. The obtained product was purified by precipitation from diethyl ether followed by vacuum filtration. Analysis with ^1^HNMR in CDCl_3_ on a Bruker AVANCE (400 MHz, Billerica, MA, USA) platform with Topspin 4.5.0 software confirmed the composition of the block copolymer: δ = 1.35–1.45 (d, CH3 in PPS chain), 2.6–2.7 (m, -CH in PPS chain), 2.85–3.0 (m, -CH2 in PPS chain), 3.38 (s, -OCH3), 3.52–3.58 (t, -OCH2CH2S), 3.5–3.7 ppm (s, broad, -OCH2CH2 in PEG chain protons), 7.8–7.83 (m, 1H, pyridine group) (Figure S1A). Every batch of newly synthesized polymer was analyzed by ^1^H NMR spectroscopy to confirm the structure and the predicted molecular weight of the PEG (2000 Da) and PPS (1440 Da) blocks. Further analysis by Gel Permeation Chromatography (GPC, Figure S1B) with a 1 mL/min DMF mobile phase settled at 55 °C and a refractive index detector (model RID-20A, Shimadzu, Kyoto, Japan) was performed to measure the polydispersity of each batch of polymer. Polymers were stored at −20 °C, where they are stable for months under inert conditions.

### 2.3. Preparation and Characterization of Drug-Integrating Amphiphilic Nanomaterial Assemblies (DIANAs)

(i) nMICs. Empty nanomicelles (nMICs) were prepared from 20 to 40 mg of the PEG_44_–PPS_20_ block copolymer using the cosolvent evaporation (CoSE) method [37]. The PEG_44_–PPS_20_ was dissolved in dichloromethane (DCM, 0.5 mL) and then added dropwise to distilled water (0.5 to 1 mL). The mixture was stirred at room temperature and left in open air for a few hours until the DCM was completely evaporated, at which point the aqueous phase contained different amounts of nMICs depending on the percentage of polymer in water used. When needed, the complete evaporation of the organic phase was achieved under vacuum.

(ii) Dexa-nMICs. Nanomicelles loaded with Dexa were obtained using a mass ratio (mg/mg) of PEG_44_–PPS_20_ block copolymer versus drug of 10 (40 mg of polymer and 4 mg of Dexa) via a slightly modified CoSE method with respect to the one used for empty nanomicelles [37]. The PEG_44_–PPS_20_ and Dexa were weighed together and dissolved in methanol (MeOH, 0.5 mL) before being added dropwise to distilled water (0.5 mL). MeOH was used for the preparation of Dexa-loaded nanomicelles because it can dissolve higher amounts of Dexa than DCM, while it is still able to resuspend the PEG_44_–PPS_20_ co-polymer. The mixture was stirred at room temperature and left in open air overnight (or for about 12 h) to make sure the MeOH was completely evaporated, at which point the remaining aqueous phase contained Dexa-loaded nanomicelles (Dexa-nMICs). When needed, the complete evaporation of the organic phase was achieved under vacuum. The sample underwent fast centrifugation at 10,000 rpm for 5 min to remove any unloaded drug.

(iii) DexP-nMICs. Nanomicelles loaded with DexP were obtained using a mass ratio (mg/mg) of PEG_44_–PPS_20_ block copolymer versus the drug of 10 (40 mg of polymer and 4 mg of DexP) via the CoSE method [37]. The PEG_44_–PPS_20_ and DexaP were weighed together and dissolved in dichloromethane (DCM, 0.5 mL), and the organic phase containing polymer and drug was added dropwise to distilled water (0.5 mL). The mixture was stirred at room temperature and was left in open air until DCM completely evaporated, at which point the remaining aqueous phase contained DexP-loaded nanomicelles (DexP-nMICs). When needed, the complete evaporation of the organic phase was achieved under vacuum. The sample underwent fast centrifugation at 10,000 rpm for 5 min to remove any unloaded drug. The supernatant was separated and stored for one night at 4 °C to ensure that all the molecules of DexP unloaded or weakly incorporated precipitated and were removed by a second fast centrifugation.

The mean diameter and polydispersity of all the nMIC preparations in aqueous media, with or without cargos, were confirmed by dynamic light scattering using a Malvern Zeta-sizer Nano Range (DLS, Zetasizer Nano ZS, Malvern Instrument Ltd., Malvern, UK), diluting the nanomicelles to have a final block copolymer concentration of about 10 mg/mL.

### 2.4. Nanomicelle Drug-Loading Efficiency

The drug-loading efficiency into nMICs was assessed by isocratic reverse-phase HPLC method (RP-HPLC) using a partisphere C18 column, 150 × 4.6 mm, on a HITACHI LaChrom Elite equipped with UV–Vis photodiode array detector (HITACHI L-2480, Tokyo, Japan). Samples prepared by diluting 20 µL of the nanoparticles with 20 µL of pure water (1:1) were injected using pure MeOH as the mobile phase for DexP-nMIC samples, or a mixture of 80 to 20 of MeOH and water for Dexa-nMIC samples, with a flow rate of 1.0 mL/min. The absorbance of DexP was quantified at a wavelength of 210 nm, with that of Dexa at 240 nm.

For each sample analyzed, a reference sample containing only the drug weighing the same as the initial drug level added to the polymers was used, dissolved in MeOH, and assessed in the same way. The average results (Table 1) are expressed as drug encapsulation efficiency (EE) and drug loading (DL), calculated as already reported and shown below:

EE(%) = amount of loaded drug/amount of added drug × 100 (1)

DL(wt/wt) = amount of loaded drug/amount of polymer carrier(2)

### 2.5. Preparation of Fluorescently Labeled nMIC

Fluorescently labeled nMICs were obtained by mixing 40 mg of PEG_44_–PPS_20_ block copolymer, previously dissolved in 0.250 mL of DCM, and 0.250 mL of the lipophilic far-red fluorescent carbocyanine dye (DiD) from a stock solution of 2.5 mg/mL in DCM (to obtain a final amount of DiD of 0.625 mg). Then, the fluorescent nMICs were prepared using a similar CoSE method to the one described for the empty nMICs. Here, the mixture of a total volume of 500 µL, containing polymer and dye, was added dropwise to 1.0 mL of distilled water. The mixture was stirred at room temperature and left in open air until DCM was completely removed by evaporation, at which point the aqueous phase contained the DiD-labeled nMICs (DiD-nMICs).

To ensure that there are no free dye molecules suspended in the solution and all the dye is entrapped in the inner core of the nMICs, the DiD-nMIC solution was centrifuged at high speed (10,000 rpm for 5 min at 5 °C). The supernatant was collected and further dialyzed at room temperature for 24 h in a Spectra/Por semipermeable membrane with Molecular Weight Cut-off (MWCO) of 3.5 kDa (Thermo Fisher Scientific, Waltham, MA, USA) against 1000 times their volume of deionized water to remove any unloaded dye molecules that escaped centrifugation. The fluorescent nMICs obtained with this process were used 100-fold diluted in all the following experiments, unless specified differently.

### 2.6. Drug Release Study

The kinetics of the release of Dexa and DexP from nMICs were studied in vitro using a dialysis method [37] to assess how fast the drugs diffuse out of their carrier. Two milliliters of Dexa-nMICs or DexP-nMICs, freshly prepared as described above (keeping the polymer/drug mass ratio of 10), were placed in a Spectra/Por dialysis membrane (Spectra Pore, Thermo Fisher Scientific) with an MWCO of 6–8 and 12–14 kDa, respectively, and incubated against 200 mL of aqueous buffer (PBS) under gentle stirring. Samples of 50 µL were collected every 24 h from the dialysis membrane. Simultaneously, the PBS against the dialysis was also refreshed every 24 h to avoid saturation with the released drug and to maintain sink conditions (dynamic exchange through the membrane). The percentage of drug released with respect to the amount initially loaded was quantified for each collected sample using RP-HPLC as described earlier. We determined the amount of either Dexa or DexP still retained in the nMICs at each time point. The release profiles were fitted with a first-order kinetic model that was used to calculate the release rate constant (*k*) and the time at which 50% of the drug content was released (half-life, *t*_1/2_). Experiments were performed three separate times.

### 2.7. Inhibition of Interleukin-6 Secretion from Mouse Macrophages In Vitro

To assess the pharmacological activity of DexP-nMICs, we evaluated their ability to suppress interleukin-6 (IL-6) secretion from stimulated macrophages using RAW 264.7 cells (ATCC, Manassas, VA, USA). Cells were seeded in a 24-well plate at a density of 200,000 cells/well. After 3 h of incubation at 37 °C and in 5% (*v*/*v*) of CO_2_, they were stimulated with 0.10 µg/mL of lipopolysaccharides (LPS), immediately followed by addition of different amounts of DexP-nMICs (from a stock formulation with DL = 0.0875 wt/wt) corresponding to concentrations of the Dexa-active molecule between 0.0062 µg/mL and 25 µg/mL. Cells treated with a water-soluble derivative of Dexa (dexamethasone disodium phosphate, DexSP, Figure 1C) at the same Dexa concentrations were used as positive controls. Cells supplemented with empty nMICs in concentrations corresponding to the amounts of nMICs loaded with DexP were used as negative controls to determine if any effect was due to the nMICs sequestering IL-6 molecules. Cells treated only with the stimulator (LPS+) were used as positive controls, while unstimulated cells (LPS-) were used to evaluate the basal level of secreted IL-6. Cells were further incubated for 24 h at 37 °C and 5% of CO_2_. After incubation, the cell supernatants were collected, and the IL-6 secretion was evaluated in each sample using a mouse IL-6 DuoSet ELISA kit (R&D Systems, Minneapolis, MN, USA). Results were normalized for total protein content using a bicinchoninic acid assay (BCA, Thermo Fisher Scientific) and expressed as percentage of the positive control. The experiment was performed in triplicate for each treatment and was repeated three times independently.

### 2.8. Inhibition of NF-kB Secretion from Human Monocytes In Vitro

THP1-Blue NF-κB cells (InvivoGen, San Diego, CA, USA) were used for monitoring the NF-κB signal transduction pathway in a physiologically relevant cell line. This cell line was derived from the human THP-1 monocyte cell line by stable integration of an NF-κB-inducible SEAP (secreted embryonic alkaline phosphatase) reporter construct. As a result, THP1-Blue NF-κB cells allow for the monitoring of NF-κB activation by assessing the activity of SEAP directly in the cell culture supernatant with QUANTI-Blue (InvivoGen, San Diego, CA, USA), a SEAP detection reagent. Cells were thawed, resuspended, and maintained as suggested by the manufacturer. For the experiment, cells were plated in a 96-well plate at a density of 100,000 cells/well and stimulated with 1.0 ng/mL of TNFα. Before stimulation, cells were pre-incubated, or not, with the treatments used above (DexP-nMICs, unloaded nMICs, and DexSP) in amounts in which the active molecule (Dexa) concentration was between 1.6 × 10^−5^ and 0.625 μg/mL. For the unloaded nMICs, the amount was calculated based on the mass of polymer present in each DexP-nMIC dose. Untreated cells were considered as positive controls. After 24 h, cell supernatants were added with Quanti-Blue (used as suggested by the manufacturer, InvivoGen, San Diego, CA, USA) to measure the NF-κB-inducible SEAP reporter construct by reading the OD at 650 nm. Results are reported as percentage of maximum expression of NF-κB-inducible SEAP, which was calculated from the positive control.

### 2.9. Perifusion Studies with Human Islets

The perifusion experiments (dynamic glucose-stimulated insulin secretion, GSIS) were performed using a PERI4 machine (Biorep Technologies, Miami, FL, USA) that allows parallel perifusion of up to 8 channels via a microfluidic manifold. Human pancreatic islet samples were procured from the Integrated Islet Distribution Program (IIDP) at City of Hope (Duarte, CA, USA). All islet samples used here were from non-diabetic donors. For each experiment, an estimated 100 IEQ of islets were handpicked and loaded in Perspex microcolumns between two layers of acrylamide-based microbead slurry (Bio-Gel P-4, Bio-Rad Laboratories, Hercules, CA, USA) by experienced operators. Perifusing buffer containing 125 mM NaCl, 5.9 mM KCl, 1.28 mM CaCl_2_, 1.2 mM MgCl_2_, 25 mM HEPES, and 0.1% bovine serum albumin at 37 °C with selected glucose or KCl (25 mM) concentrations was circulated through the columns at a rate of 100 μL/min. After 60 min of washing with low-glucose (4 mM, G4) solution for stabilization, islets were stimulated with the following sequence: 8 min of low glucose, 25 min of high glucose (16 mM, G16), 15 min of low glucose, 10 min of KCl, and 20 min of low glucose. Samples (100 μL) were collected every minute from the outflow tubing of the columns in an automatic fraction collector designed for a multi-well plate format. The islets and the perifusion solutions were kept at 37 °C in a built-in temperature-controlled chamber, while the perifusate in the collecting plate was kept at <4 °C to preserve the integrity of the analytes. Insulin concentrations were determined with commercially available human ELISA kits (Mercodia Inc., Winston Salem, NC, USA). Values obtained with the human kits were converted from mU/L to μg/L using 1 μg/L = 23 mU/L per the manufacturer’s guidelines. Because accurately assessing islet mass in islet equivalent (IEQ) units is nontrivial [49], to account for possible differences among islets in different channels, values were adjusted based on the response to KCl using the area under the curve (AUC) in each column for normalization as described before [50,51]. All responses are scaled to 100 IEQ.

### 2.10. Animal Work

All studies involving animal subjects were performed under protocols approved and monitored by the University of Miami Institutional Animal Care and Use Committee (IACUC; protocol 22-136). All procedures were conducted according to the guidelines of the Committee on Care and Use of Laboratory Animals, Institute of Laboratory Animal Resources (National Research Council, Washington, DC, USA). All animals were obtained from Jackson Laboratories (Bar Harbor, ME, USA) and housed at the Division of Veterinary Resources, University of Miami (Miami, FL, USA).

#### 2.10.1. Pharmacokinetic Study in Mice and Rats

The pharmacokinetic (PK) properties of the DexP-nMIC formulations were studied in mice (C57BL/6) and rats (Lewis). All formulations were administered at equivalent concentrations of the active compound (Dexa) and at a concentration of PEG_44_–PPS_20_ block copolymer (40 mg/mL) >100 times higher than its critical aggregation concentration (0.35 mg/mL [37]). The blood level of Dexa was measured using a highly sensitive forensic ELISA kit (Neogen, Lansing, MI, USA), which can detect amounts as low as 0.02 nM, used as suggested by the provider.

C57BL/6 mice were divided into four treatment groups: 1. Control, which received no treatment (*n* = 5); 2. DexSP (*n* = 6); 3. Dexa-nMICs (*n* = 5); and 4. DexP-nMICs (*n* = 6). Treatments were administered systemically via intravenous (IV) injections in the tail vein at time = 0, and doses were determined to contain 8.75 mg/kg of Dexa. Blood samples were collected 1 h before the injections to measure the baseline and at 1, 6, 24, 48, 72, 96, and 120 h after the injections.

A similar study was performed in Lewis rats using the same treatment groups used for the mice but with a different dose of Dexa (4.45 mg/kg): 1. Control (*n* = 4); 2. DexSP (*n* = 4); 3. Dexa-nMICs (*n* = 6); and 4. DexP-nMICs (*n* = 6). Samples were administered systemically via retro-orbital (RO) injections at time = 0, and blood samples were collected 1 h before the injections (to measure the baseline) and at 1, 6, 24, 48, 72, and 96 h after the injections.

#### 2.10.2. Mouse Allogenic Skin Transplant Model

An allogenic skin transplant was performed in mice to assess the therapeutic potential of systemically administered DexP-nMICs and compare it with that of free Dexa (used as its soluble derivative DexSP). This study was performed using BALB/C mice as skin donors and C57BL/6 as recipient mice. The recipient mice were divided into three groups: 1. Control (untreated, *n* = 9); 2. DexSP (10 mg/kg, *n* = 5); and 3. DexP-nMICs (12 mg/kg; *n* = 6). The amount of active molecule was equal to 7.5 mg/kg Dexa in each treated group, and the concentration of PEG_44_–PPS_20_ block copolymer > 100 times higher than its critical aggregation concentration, as for the PK study (Section 2.10.1). Treatments started one day before transplant and were repeated on post-operation day (POD) 1 and continued every 72 h until POD 15. Treatments were administered systemically via intraperitoneal (IP) injections.

(i) Donor skin harvest. Donor mice were anesthetized with isoflurane (induction vaporizer at 4%, maintenance at 1–2% through the mouse cone). Then, using sterile scissors, the skin grafts were harvested from the ears, and the animals were euthanized by carbon dioxide asphyxiation followed by cervical dislocation. Under a microscope, the connective tissue, fat tissue, and panniculus carnosus were separated from the back skin using fine tenotomy scissors. The grafts were stored for a short time on gauzes soaked with sterile phosphate-buffered saline (PBS) on ice. Two grafts were obtained from each donor mouse.

(ii) Recipient skin transplant. The recipient mice were anesthetized with isoflurane (induction vaporizer at 4%, maintenance at 1–2% through the mouse cone), shaved on the side of the back where the graft was to be inserted, and disinfected with 10% povidone iodine. Using scissors, the skin was gently lifted, and a small piece was superficially cut, creating an oval-shaped opening of approximately 100 mm^2^ in size. The size was selected to be slightly larger (10%) than the graft and was cut as superficially as possible to preserve the panniculus carnosus and vessels. The previously prepared graft was then positioned on the graft bed avoiding folds along the edges. Finally, the recipient mice were wrapped into adhesive bandages. The animals were monitored closely during recovery to ensure that the bandages were not restricting thorax excursion and breathing.

(iii) Evaluation of skin-transplant survival. Seven days after surgery, the bandages were removed under anesthesia. The grafts were closely inspected visually and palpated for signs of scabbing, contraction, or hardness. Cases where the graft did not achieve proper vascularization were considered a technical failure. Otherwise, on the following days, the grafts were monitored daily for signs of rejection. The grafts were considered rejected when 90% of the graft tissue became necrotic.

(iv) Mouse skin transplant live imaging. Fluorescently labeled nMICs (DiD-nMICs) were used in a whole body In Vivo Imaging System (IVIS; Revvity, Waltham, MA, USA) to determine the distribution of the nMICs into the skin graft. Specifically, 50 µL of DiD-nMIC stock solution was administered to mice that had undergone skin transplantation but were not treated with any drug formulation. The DiD-nMICs were administered on POD 8, after the bandage had already been removed. Mice were imaged using the IVIS 24 and 96 h after the injection of DiD-nMICs. Immediately after in vivo imaging at 96 h, mice were euthanized and the skin and the major organs were collected and imaged ex vivo using the IVIS. Fluorescence intensity of DiD-nMICs in live animals was quantified using the Living Image Software 4.8.2 (Revvity, Waltham, MA, USA), and ROI (region of interest) analysis was performed to estimate the fluorescent intensity of the DiD-nMICs in the skin graft and the area in the vicinity of the graft (assessed as the average of four areas of similar sites surrounding the graft) 24 and 96 h post-injection. A mouse bearing a skin graft that did not receive the DiD-nMIC injection was used as control to set the fluorescence baseline. Fluorescence intensity of the DiD-nMICs in the explanted organs was also evaluated ex vivo using the ROI method.

Generative artificial intelligence (GenAI) has not been used in any part of this paper.

## 3. Results and Discussion

### 3.1. Synthesis and Self-Assembling of PEG_44_–PPS_20_ Block Copolymers

The diblock copolymer PEG–PPS was designed and synthesized as material for developing biocompatible nanoparticles able to achieve controlled drug delivery. Within this family, the PEG_44_–PPS_20_ block copolymer with *f*_PEG_ 0.57 (Figure 2A) was selected because in water it self-assembles, forming solid core nanomicelles (nMICs, Figure 2B) that can load high amounts of immunomodulatory drugs [30]. In general, the synthesis of PEG-PPS, performed by an anionic living polymerization of propylene sulfide [37,52] in mild conditions, allows very good control over the episulfide degree of polymerization with high product recovery (about 80–90% of the initial weight mass) [30,36]. The PEG block improves biocompatibility and solubility and allows nanoparticle resistance to protein adsorption [53]. The poly-propylene sulfide (PPS) is an oxidation-sensitive, low-glass-transition-temperature, and hydrophobic polymer, used because of its advantage of turning water-soluble once oxidized in the cellular environment, making it susceptible to easy elimination from the body. As a result of their amphiphilic composition, PEG−PPS self-assemble in water forming mesophases, with the shape and size determined by the mass ratio of the two blocks [52]. Therefore, we could predict with confidence that the PEG_44_–PPS_20_—with MW_PEG_ = 2000 Da and MW_PPS_ = 1440 Da, confirmed by ^1^HNMR, and PDI = 1.13 ± 0.045, confirmed by GPC analysis (Figures S1A and S1B, respectively)—prepared for this work will form homogeneous nMICs of diameter within the 20–25 nm range (Figure 2B). Among other mesophases, the PEG_44_–PPS_20_ nMICs demonstrated the highest drug-loading efficiency, longest sustained-release capability of hydrophobic drugs, and best stability and biocompatibility [30]. Therefore, we consider these nMICs an ideal system for delivery of immunosuppressive and/or anti-inflammatory drugs, such as Dexa and its derivative, in the context of cell and tissue transplantation.

The PEG_44_–PPS_20_ block copolymers synthesized here (Figure 2A) were self-assembled in water by modifying the cosolvent evaporation method already reported for nMICs (Figure 2B), either loaded or not with the cargo drug (Dexa or DexP). Dynamic light scattering (DLS) confirmed a diameter of about 25 nm for unloaded nMICs and for DexP-loaded nMICs (Figure 2C). Despite the relatively small size (~25 nm), the tight hydrophobic PPS core allowed efficient and stable loading of hydrophobic drugs. For example, for Dexa, a drug/polymer mass ratio of 1 to 10 (Dexa 5.0 mg / PEG_44_–PPS_20_ 50 mg) resulted in 34.06% of the total Dexa mass loaded into nMICs (Table 1). For DexP, a drug/polymer mass ratio of 1 to 10 (DexP 4.0 mg / PEG_44_–PPS_20_ 40 mg) resulted in 58.35% of the total DexP mass loaded into nMICs (Table 1). Given these results, we can estimate that compared to the most used polymer systems (e.g., Pluronic F127 or PEG-PLGA [54]), nMIC DIANAs provide higher drug solubilization in aqueous media for Dexa, considerably increasing the solubility of this drug that is practically insoluble in water.

Furthermore, HPLC analysis performed after storing the formulations at 4 °C for several days confirmed that the amount of drug loaded in the nMICs is stable over time. Thus, drug-loaded nMICs do not need to be prepared freshly for each experiment. If stored at 4 °C under inert conditions (argon gas) PEG_44_–PPS_20_ nMICs can last for months without releasing their payload, which is particularly attractive for prospective clinical applications.

### 3.2. Drug Release

Characterization of the drug release profile is crucial because it determines how long the drug remains stable protected by its carrier, how frequently it needs to be administered, and whether the carriers can provide a sustained therapeutic effect. The release kinetics of Dexa and DexP from nMICs were studied in vitro for both the prodrug (DexP) and the active molecule (Dexa). Experiments were performed using the dialysis technique as described previously for cyclosporine A [30], mimicking the in vivo environment by applying sink conditions. This method avoids the risk of saturating the dialysis buffer once the drug is released from the nMICs, which could compromise its diffusion.

**Figure 3:**
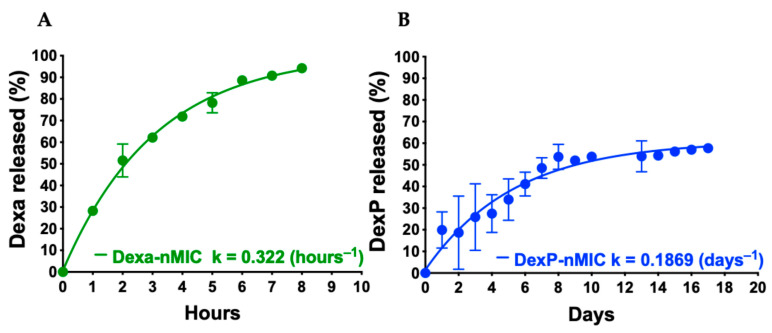
Release kinetics of dexamethasone Dexa (**A**) and dexamethasone palmitate, DexP, (**B**) from nMICs. The release study was performed via a dialysis method using a 6–12 kDa MWCO membrane against distilled water and quantified by RP-HPLC (*n* = 3). The release rate constants (*k*) and half-lives (*t*_1/2_) calculated by fitting a first-order release model are included on the graphs.

Results showed that while Dexa diffuses out of the nMICs within a few hours (Figure 3A), DexP is retained in the nMICs much longer due to its more hydrophobic chemical structure (Figure 1), and only a small percentage diffuses out in 24 h (Figure 3B). This is because the palmitate chains strongly interact with the hydrophobic core of the nanomicelles made by the self-aggregated PPS chains. Therefore, our nMICs can achieve controlled and prolonged DexP release, which can help to reduce the frequency of administration, minimizing fluctuations in drug concentration. This sustained-release profile enhances therapeutic efficacy and safety, making it a promising strategy for improving drug delivery and optimizing treatment outcomes.

Detailed analysis of our results shows that the release profile of DexP from nMICs exhibited a sustained and controlled release pattern over approximately two weeks, its cumulative release reaching a plateau at around 60% of the loaded dose (Figure 3B). The data were fitted with a classic first-order release model, which is commonly used to describe the release kinetics of drugs from various delivery systems including nanoparticles [55], and suggested a release rate constant (*k*) of 0.1869 day^−1^ corresponding to a half-life (*t*_1/2_) of ∼3.7 days (89 h). The initial phase showed a moderate burst release, likely due to loosely bound DexP at the micelle interface, followed by a more controlled and gradual release phase indicative of a diffusion-driven mechanism. The release kinetics of DexP-nMICs (Figure 3B) was significantly more favorable than that of Dexa-nMICs (Figure 3A). Dexa is released from the nMICs within only a few hours (*t*_1/2_ ∼2.17 h), leading to a more rapid dispersion and consumption through the organism.

The observed kinetics suggest that the nMICs explored here enable a sustained and well-regulated drug release profile, ensuring a gradual and prolonged delivery of the Dexa prodrug DexP. Once released, DexP must be hydrolyzed to convert into its pharmacologically active form, Dexa, possibly further increasing the sustained nature of its action. Therefore, we anticipated a more prolonged circulation of the drug in the bloodstream, maintaining consistent therapeutic levels over longer periods of time and reducing the frequency of dosing, ultimately improving patient compliance and overall clinical outcomes.

### 3.3. Inhibition of Interleukin-6 Secretion In Vitro Using Mouse Macrophages

**Figure 4:**
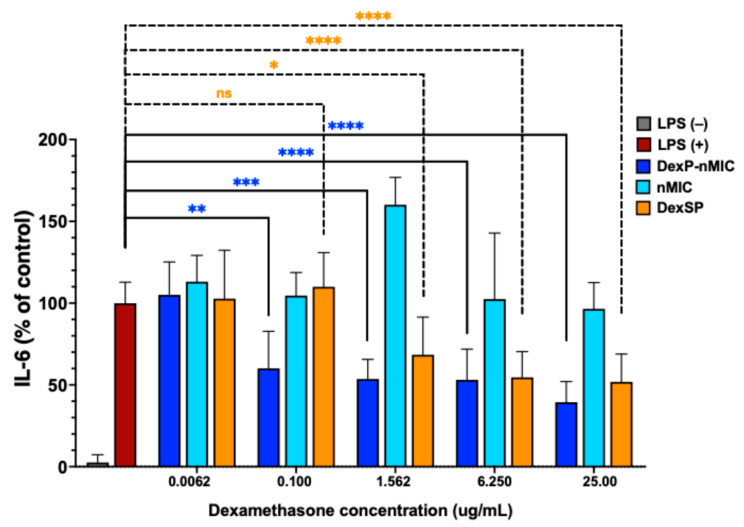
Inhibition of IL-6 cytokine secretion in LPS-stimulated RAW 264.7 macrophages by DexP-nMICs (blue bars) as compared to empty nMICs (cyan bars) and free DexSP (orange bars) used at dosages corresponding to the same concentrations of the active molecule Dexa. Asterisks indicate statistically significant differences, ns indicates non-significant differences compared to the LPS-stimulated cells (unpaired *t* test; * *p* < 0.05, ** *p* < 0.01, *** *p* < 0.001, **** *p* < 0.0001, *n* = 3).

Next, it was critical to prove that DexP-nMICs can provide pharmacologically active Dexa. This was performed by evaluating the inhibition of interleukin-6 (IL-6) secretion from activated macrophages after treatment with various concentrations of DexP-nMICs. IL-6 is a key pro-inflammatory cytokine involved in the immune response, playing a crucial role in the regulation of inflammation, infection control, and tissue injury repair; however, excessive IL-6 production is associated with various chronic inflammatory diseases, including T1D. Furthermore, during the first period after allograft implantation, macrophages are recruited to the allograft from circulation as monocytes and can also proliferate from resident macrophages within the graft. These recruited macrophages can contribute to graft injury by promoting inflammation, tissue damage, and the release of inflammatory mediators. Therefore, testing the ability of DexP-nMICs to suppress the IL-6 production in vitro allowed us to assess whether DexP-nMICs can enhance the cellular uptake and, thus, the bioactivity of Dexa compared to the water-soluble form of Dexa (unformulated DexSP). Figure 4 shows the percentages of IL-6 secreted from LPS-stimulated RAW 264.7 macrophages after treatment with different doses of DexP-nMICs (blue columns), free DexSP (orange columns), or empty nMICs (negative controls, cyan columns). Unstimulated macrophages (gray column) show the basal level of cellular IL-6, which is minimal as expected, while LPS-stimulated macrophages (dark red, positive controls) exhibit a dramatic increase in IL-6 production, confirming the inflammatory response induced by LPS on the RAW 264.7 cell line.

This experiment provided interesting insights into the anti-inflammatory efficacy of the nMIC formulation of DexP with respect to the free form of the drug. A notable trend is that DexP-nMIC formulations (Figure 4, blue columns) already inhibited about 50% of IL-6 secretion at the dose corresponding to 0.10 µg/mL of free Dexa, while DexSP needed a higher dose to be similarly effective (Figure 4, orange columns). This can be due to the higher stability of Dexa formulated as DexP-nMICs and to a more efficient cellular internalization with respect to the free form of the drug. Although further increases in the concentration of DexP-nMICs (corresponding to Dexa concentrations between 1.56 µg/mL and 25 µg/mL) only slightly increased the effect on IL-6 secretion, the effect remained significant (Figure 4, blue asterisks). There was a trend showing concentration dependency, as indicated by the increasing statistical significance levels at higher DexP-nMIC concentrations (Figure 4, blue columns). This supports the theory that DexP is slowly released and hydrolyzed in a controlled manner from the nMICs, and that DexP-nMICs had already saturated the macrophages within 24 h at low concentration, thus larger amounts are not necessary. Indeed, all tested concentrations of DexP-nMICs (corresponding to Dexa contents of 0.10, 1.56, 6.25, and 25 µg/mL) significantly reduced IL-6 secretion by at least 50% compared to the LPS-stimulated cells used as positive control, but the amount of DexP-nMICs corresponding to a concentration of active drug of 0.10 µg/mL was already sufficient to release a clinically effective intracellular concentration of Dexa. Note also that the empty nMICs did not inhibit IL-6 production, indicating that the observed effect is due to the intracellular release of DexP and the nanocarrier had no significant effect on IL-6 secretion (Figure 4, cyan columns).

Overall, these findings proved the clear advantage of using nMICs as drug carriers, and confirmed that DexP-nMICs enhance drug uptake and efficacy in just 24 h and can successfully deliver Dexa in its biologically active form leading to a robust suppression of IL-6 secretion.

### 3.4. Inhibition of the NF-κB Pathway in Human Monocytes

**Figure 5:**
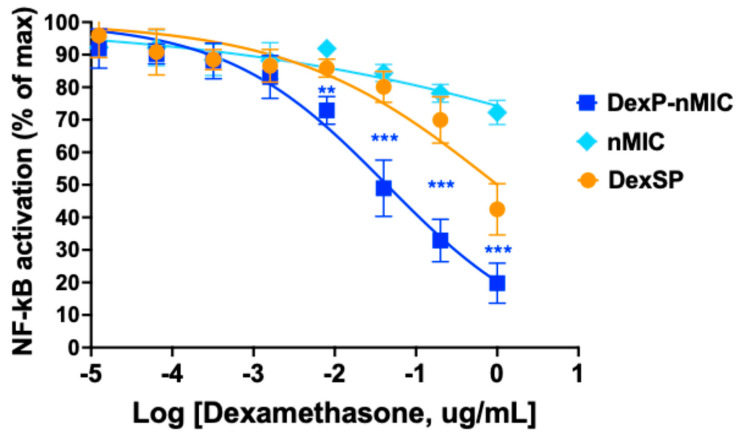
Inhibition of TNFα-induced NF-κB activation in THP-1 monocytes. THP1-Blue NF-κB cells were activated with TNFα and treated with DexP-nMICs (blue), empty nMICs (cyan), and free DexSP (orange) used at concentrations corresponding to the same concentrations of the active molecule Dexa. Curves show the concentration-dependent response on NF-κB activation for each treatment. Asterisks indicate statistically significant differences between the effects of DexP-nMICs and DexSP at the corresponding concentrations as used in this experiment (equivalent with Dexa 0.005, 0.025, 0.125, and 0.625 μg/mL) (multiple Mann–Whitney tests; ** *p* < 0.01, *** *p* < 0.001, *n* = 6).

We also tested the anti-inflammatory activity of DexP-nMICs and compared it to that of DexSP in human monocytes. As shown in Figure 5, the suppression of TNFα-induced NF-κB activation was significantly more efficient in THP-1 cells treated with DexP-nMICs (blue line and squares) than in those treated with DexSP (orange line and circles), confirming that the concentration of Dexa needed for bioactivity is lower if the drug is formulated as DexP-nMICs. Asterisks in Figure 5 indicate statistically significant differences between treatment with DexP-nMICs and DexSP at the amounts used in this experiment corresponding to the concentration range 0.005–0.625 μg/mL of Dexa. The graph also indicates a clear concentration-dependent response with half-maximal reduction in the NF-κB activation at 0.025 μg/mL, four times lower than needed in the previous macrophage cell assay. As before, empty nMICs did not have a significant effect (Figure 5, cyan). These results confirm again that DexP-nMICs are pharmacologically more efficient in vitro than free DexSP, making them a promising system for an LISAI regimen.

### 3.5. Effect on GSIS in Human Islets

**Figure 6:**
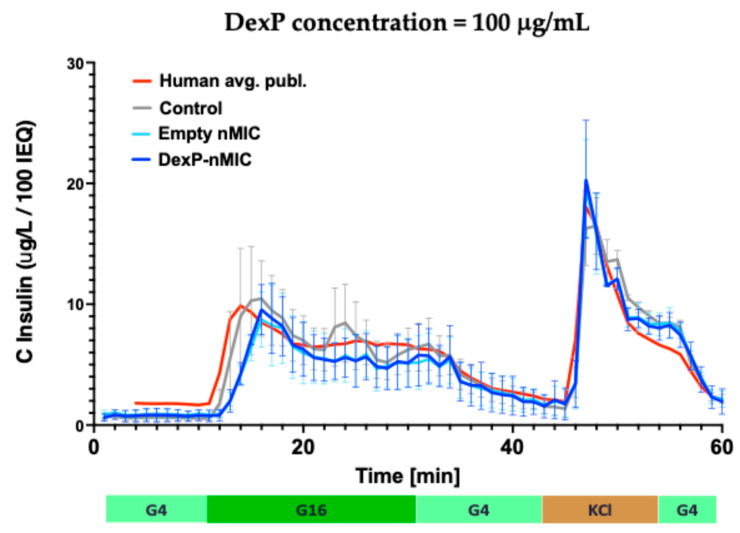
Dynamic perifusion assays confirming that the DIANAs used here, including unloaded (empty), nMICs (cyan), and DexP-nMICs (blue), do not affect the insulin secretion of human islets at the concentration tested. Untreated islets were used as control (gray), and data shown in red are the average of multiple human islet samples, as reported previously [51] and included here for reference. Results shown for dynamic insulin secretion after 24 h of incubation from islets stimulated using an automated multichannel perifusion apparatus and a standard protocol of low (4 mM; G4, 8 min), high (16 mM; G16, 20 min), and low (4 mM; G4, 15 min) glucose stimulation followed by KCl and G4 (each for 10 min) as shown below the graph. Output samples were collected every minute at 0.1 mL/min flow rate and ~100 IEQ per channel. Data are average ± SD for *n* = 2 human islet samples.

A major concern of using glucocorticoids such as Dexa and its derivatives in pancreatic islet transplantation is their potential toxicity on insulin-producing beta cells (including potential inhibition of insulin release) as that can be an issue even with local administration. On the other hand, glucocorticoids may in fact enhance insulin secretion in islets [56,57], and they have overall beneficial effects on islet transplantation and function by reducing the effects of inflammatory cytokines [58,59]. We have already confirmed that the nMICs and nFIB DIANAs themselves do not affect the viability and function of islets at concentrations needed for LISAI treatments [30]. Here, we tested the DIANAs used here (nMICs and DexP-nMICs) on human islet cells using dynamic perifusion assays to evaluate in detail their effect on glucose-stimulated insulin secretion (GSIS). Perifusion assays characterize the entire time profile of insulin secretion under controllable conditions of glucose influx and, thus, are the most complex in vitro assay to assess the function of isolated islets. They can provide more sensitive assessments and more detailed information than the more widely used static GSIS assay [51]. These dynamic perifusion assays confirmed that the empty and the cargo-loaded DIANAs used here are non-toxic and do not affect insulin secretion of human islets as the insulin secretion time profiles were not affected by incubation with these nMICs (Figure 6). While free Dexa has been shown to suppress insulin secretion in perifusion studies (e.g., at 1000 nM after 3 h pre-incubation [60] or possibly even at 40 nM after 24 h pre-incubation [61]), DexP-nMICs had no effect here.

### 3.6. Pharmacokinetic (PK) Study in Mice and Rats

**Figure 7:**
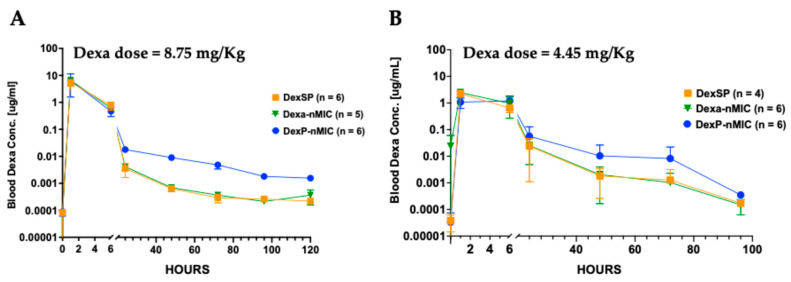
Pharmacokinetic (PK) studies in mice (**A**) and rats (**B**) showing blood Dexa levels after systemic administration (IV) of various treatments as indicated (note the large range of the vertical axis showing concentrations on log-scale). (**A**) PK studies in mice, showing that DexP-nMICs (blue) prolong the circulation time of the active Dexa as compared to free Dexa (DexSP, orange) or Dexa-nMICs (green) administered at equivalent concentrations of the active drug (Dexa, 8.75 mg/kg). (**B**) PK studies showing the same in rats at equivalent concentrations of the active drug (Dexa, 4.45 mg/kg). Blood Dexa concentration data shown were quantified using a sensitive ELISA kit (lowest limit of detection 0.02 nM); *n* = 4 to 6 animals per group as indicated on the graphs.

A key challenge in pharmacotherapy is maintaining optimal drug concentration over time. Many drugs currently in use, including Dexa, although very potent, suffer from a short half-life, requiring frequent dosing. Therefore, we prioritized investigating the PK properties of DexP-nMICs with respect to those of the free drug (DexSP) as well as the active molecule itself formulated with nMICs (Dexa-nMICs). This study focused on evaluating the duration of the active drug in systemic circulation and was performed in both mice and rats. Treatments were administered via systemic injections (IV) at doses containing an equivalent amount of the Dexa active molecule (8.75 mg/kg for mice and 4.45 mg/kg for rats), as indicated in Figure 7.

Our drug delivery strategy using nMIC DIANAs is expected to improve drug solubility, reduce the administered dose, prolong drug stability, improve targeting, and enable sustained release. Indeed, PK profiles obtained in both mice (Figure 7A) and rats (Figure 7B) indicate that while Dexa-nMICs had no effect, DexP-nMICs significantly prolonged the systemic circulation of the active molecule (Dexa) compared to DexSP. In mice, DexSP (Figure 7A, orange line) and Dexa-nMIC (Figure 7A, green line) groups showed a rapid decline in blood Dexa level within the first 6 h, followed by a less steep reduction, and reaching near-baseline levels by 72 h. In comparison, the mice that received DexP-nMICs (Figure 7A, blue line) exhibited a more gradual decline during the first 6 h and maintained a detectable Dexa blood level up to 120 h, confirming that DexP-nMICs were able to provide sustained drug release.

PK was also assessed in Lewis rats, and a similar trend was observed. DexP-nMICs led to prolonged Dexa circulation compared to the other formulations, with a slower decline in blood Dexa concentration beyond 6 h (Figure 7B). Note also that on days 2–3 after administration, Dexa blood concentrations were about 10-fold higher after DexP-nMIC administration than after Dexa-nMIC or DexSP administration in both mice and rats (Figure 7).

The higher hydrophobicity of DexP allows a more efficient and more stable loading into the nMIC core and provides a more sustained release compared to the less hydrophobic Dexa, resulting in a more gradual decline in systemic drug concentrations. This enhanced PK profile, promoted by sustained release, reduces the frequency of drug administration, which is a key factor in improving therapeutic efficacy with fewer side effects.

### 3.7. Allogeneic Mouse Skin Transplant Model

Murine full-thickness skin transplantation is a well-established in vivo model for studying alloimmune responses and graft rejection. Although its direct application to humans is limited, this model is widely used in allogeneic transplantation research [62]. The advantages of this model include the highly reproducible immune response that leads to graft rejection, which can be easily monitored through visual inspection and palpation, and the relatively short duration of the study (15 to 20 days) as skin transplants are among the most stringent transplants, known to generate more aggressive immune responses than, e.g., liver, heart, and kidney transplants [63].

#### 3.7.1. Mouse Skin Transplant Live Imaging

Allogeneic mouse skin transplant was used here as a model because we have shown before that nMICs can target inflammation sites including transplant sites via passive targeting thanks to their small size (20–25 nm in diameter) [36], and because we noticed that they were particularly effective in targeting scar tissue following surgeries in mice. Indeed, this has been confirmed here by whole-body in vivo and ex vivo imaging system (IVIS) analysis using fluorescently labeled nMICs (DiD-nMICs) in a skin graft model.

**Figure 8:**
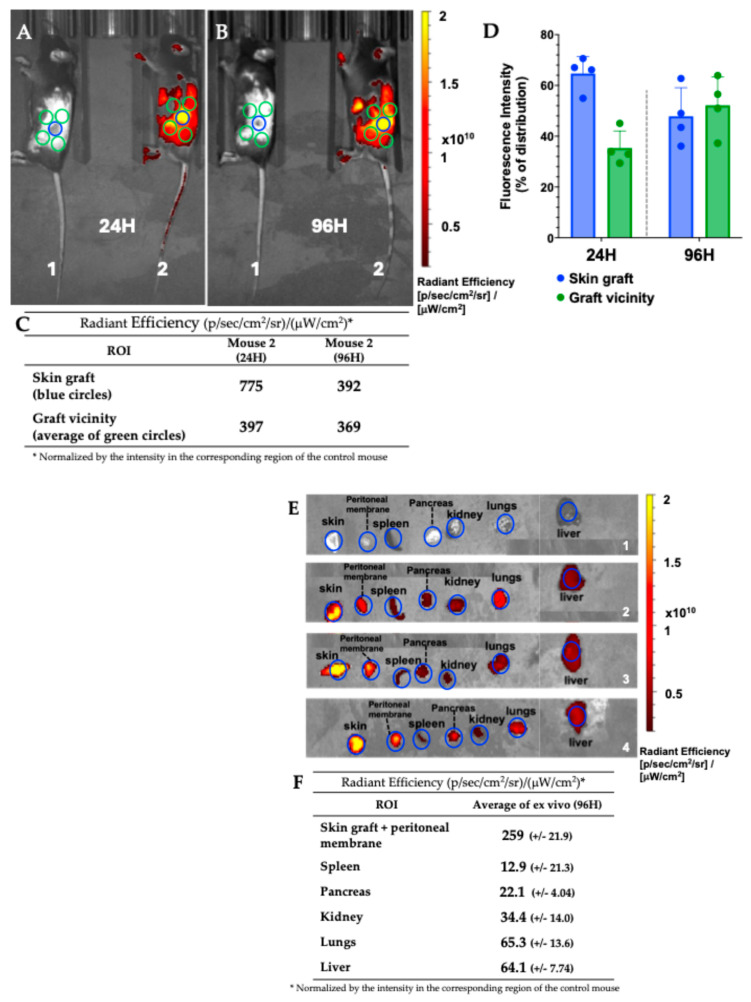
In vivo biodistribution of DiD-nMICs in the skin graft site. Representative whole-body imaging of an untreated mouse bearing a skin graft (mouse 1) and of a mouse bearing a skin graft and treated with DiD-nMICs (mouse 2) at 24 h (**A**) and 96 h (**B**) after administration (IP injection, 50 μL). Fluorescence was assessed for regions of interest (ROIs) representing the area of the skin graft (blue circles) and the vicinity of the graft (average of four green circles in the vicinity of the graft with identical dimensions). (**C**) ROI analysis of the fluorescent intensity of the skin graft (blue circles on mouse 2 in (**A**) and (**B**)) and the graft vicinity (average of green circles on mouse 2 in (**A**) and (**B**)), both normalized by the fluorescence background of the control areas (mouse 1 in (**A**) and (**B**); blue and green circles, respectively). (**D**) Percentage of the fluorescent intensity in the skin graft (blue columns) and graft vicinity (green columns) at 24 and 96 h after DiD-nMIC administration, calculated from the ROI analysis in (**C**) (*n* = 4 animals per experiment). (**E**). Ex vivo IVIS imaging of explanted organs from control mouse that was not injected (panel 1) and mice 96 h after nMIC-DiD IP injection (panels 2–4). (**F**) Ex vivo ROI analysis (blue circles in (**E**)) of the fluorescent intensity of the explanted skin and major organs (*n* = 3 animals per experiment).

Selected mice bearing a skin transplant received 50 µL of DiD-nMICs via intraperitoneal (IP) injection on POD 8, after the protective bandages were removed, while another transplanted but untreated mouse was used as control. The distribution of the fluorescent nMICs was traced by whole-body imaging (IVIS) at the 24- and 96-hour post-injection time points. This revealed clear accumulation of the nanomicelles at the site of and around the skin graft (as shown in Figure 8A and Figure 8B for a representative mouse, and Figures S2A and S2B for *n* = 4 animals). ROI (Region of Interest) analysis was performed to estimate the fluorescent intensity of the DiD-nMICs in the skin graft (Figure 8A,B, blue circles, and corresponding values in Figure 8C) with respect to the vicinity of the graft (Figure 8A,B, average of green circles, and corresponding values in Figure 8C) both 24 h (Figure 8A) and 96 h (Figure 8B) post-injection. Assuming the total fluorescence intensity as the sum of the two ROI intensities (blue and green areas), >66% of the DiD-nMICs were distributed in the skin graft, and the rest ~ 33% in the vicinity of the graft after 24 h, indicating efficient targeting (Figure 8D, left side).

Even after 96 h, >47% of the total intensity signal of DiD-nMICs was still detected in the graft, while >52% was in the vicinity, as shown in the graph of Figure 8D (right side). This diffusion of the nMICs from the graft to the vicinity occurred probably due to the expansion of the inflamed area around the graft, triggered by the rejection process, which could be beneficial for an LISAI regimen around the graft site.

Ex vivo imaging of explanted organs at 96 h after injection showed that most of the nMIC-DiD fluorescence is still accumulated in the skin graft and in the peritoneal membrane tissue below the graft (Figure 8E). The analysis of the ROI (average of the blue circles in Figure 8E) is reported in Figure 8F, and confirmed that the fluorescence intensity in the skin is significantly higher than in the other organs (e.g., 10 times higher compared to spleen, pancreas, and kidney, and almost 3 times in respect to the largest organs, such as lungs and liver).

Further optimization of our nanoparticle size, morphology, and concentration is being explored to reduce any potential accumulation outside of the inflammation site.

Overall, in vivo live imaging confirmed that the nMIC formulations not only enhanced the solubility, stability, and efficacy of their cargo drug, but also provided sustained release and preferential targeted delivery to the inflammation and transplant sites.

#### 3.7.2. Allogeneic Mouse Skin Transplant Survival

**Figure 9:**
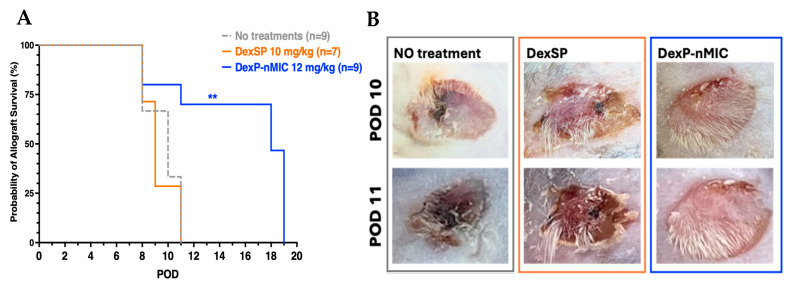
(**A**) Kaplan–Meier survival curves for allogeneic skin transplants (skin patch from the ear of BALB/C mice transplanted on the back of C57BL/6 mice). Transplanted mice were treated with DexSP (orange line) and DexP-nMICs (blue line) at equivalent concentrations corresponding to 8.5 mg/kg active drug (Dexa) starting at POD 1 and continuing every 72 h until POD 15. Untreated mice were used as controls (gray dotted line); the number of animals for each treatment group is indicated in the graph. Asterisks denote statistically significant differences compared to the untreated group (log rank Mantel–Cox test, ** *p* < 0.01). (**B**) Representative images of skin grafts at POD 10 and POD 11 in treatment groups, as indicated. In the DexP-nMIC group (blue box), no signs of rejection (black coloration, swelling, and tissue necrosis) were present, and the skin graft remained undamaged for longer than in the other mice.

Results of our allogeneic skin transplant experiment are shown in the survival curves reported in Figure 9A, highlighting the impact of different treatments on survival in a highly stringent model using BALB/C as donor mice and C57BL/6 mice as recipients. As expected, mice that did not receive any treatment (gray dashed line) experienced rapid graft rejection, with failure occurring at a median of post-operation day (POD) 10. Mice treated with DexSP (orange line) showed no significant difference in graft survival, with full rejection happening with a median of POD 9. This result suggests that the dose and the administration frequency used here for free Dexa were not enough to efficiently target the site of transplant and provide long-term anti-inflammatory and immunosuppressive effects. However, when Dexa was delivered as DexP-nMICs (blue line), the results were notably different. This group showed a statistically significant improvement in graft survival compared to the untreated mice (** *p* < 0.01), with most of the transplants remaining viable at least up to POD 18–19 (after which the graft is fully integrated in the host skin and covered by furs). This proves that DexP-nMICs provided sustained delivery of Dexa and robust inflammatory- and immune-modulation at the site of transplant.

Figure 9B corroborates these conclusions via representative images from each treatment group taken for visual assessment of the graft survival at critical time points (POD 10 and 11). It is clearly visible that for the control and DexSP treatment, the grafts were rejected (black coloration and swelling indicating tissue necrosis), whereas in DexP-nMIC-treated mice, the grafts remained intact and continued their integration within the surrounding tissue. Additional images are reported in Figure S3.

These findings are particularly important considering the challenging nature of skin transplants [63]. The ability of DexP-nMICs to significantly prolong graft survival underscores the potential of nanoparticle-based immunosuppressive strategies.

## 4. Conclusions

Pancreatic islet transplantation represents a promising therapeutic approach for T1D, but its widespread clinical application remains hindered by two critical challenges: the limited availability of donor islets, and the need for chronic systemic immunosuppression. Here, we continued to explore our Drug-Integrating Amphiphilic Nano-Assemblies (DIANAs) approach as an innovative strategy to achieve localized immunomodulation, thereby enhancing islet engraftment while reducing systemic toxicity. The findings presented in this work demonstrate that DIANA nanomicelles (nMICs) can efficiently encapsulate and sustain the release of an anti-inflammatory prodrug (DexP), providing targeted delivery to key sites involved in immune-mediated graft rejection. In vitro and in vivo experiments confirmed the potential of these nanostructures to mitigate inflammatory responses, prolong graft survival, and enhance the overall effectiveness of transplantations.

The use of PEG_44_–PPS_20_ nMICs to solubilize and deliver dexamethasone palmitate is particularly promising as the resulting DexP-nMICs can carry elevated amounts of dexamethasone, increasing its solubility in water and enhancing its pharmacokinetics in vivo.

DexP-nMICs allowed the sustained and localized release of Dexa, reducing cytokine secretion by activated mouse macrophages and human monocytes in vitro and improving graft survival in a mouse allogeneic skin transplant model in vivo compared to the unformulated parent drug.

Among the others, we have prioritized the use of very small nanomicelles because, while they still escape renal filtration, for which the size cutoff is only 5.5 nm [64], they are capable of passive targeting via the enhanced permeability and retention effects of inflamed tissues. Small nanoparticles extravasate faster and are immediately retained in the inflammation by the infiltrating macrophages. The smaller the size of the nanoparticles, the greater their penetration and retention in macrophages. In tumor models, it has been demonstrated that 100 nm nanoparticles remain close to vasculature, whereas 20 nm nanoparticles distribute better throughout the tissue and extracellular matrix [65].

Indeed, the results of this study highlight the applicability of 25 nm PEG_44_–PPS_20_ nMICs as a drug delivery system in transplantation medicine, offering a potential alternative treatment (such as an LISAI regimen) or a complementary approach to conventional immunomodulatory therapies. By reducing or avoiding the need for systemic immunosuppression, our DIANA approaches could allow more patients, including children, to benefit from transplant procedures without the high risks associated with traditional treatments.

## Figures and Tables

**Table 1:** Encapsulation efficiency (EE) and drug loading efficiency (DL) of Dexa and DexP in nMIC DIANAs. Results are expressed as mean ± standard deviation (*n* = 10).

Drug	Drug/Polymer Initial Ratio (mg/mg)	EE (%), Mean ± SD	DL (wt/wt), Mean ± SD
Dexamethasone (Dexa)	1:10	34.06 ± 8.56	0.0346 ± 0.008
Dexamethasone palmitate (DexP)	1:10	58.35 ± 9.69	0.058 ± 0.009

## Data Availability

The raw data supporting the conclusions of this article will be made available by the authors upon request. Data are stored and shared through hardware and software existing in the labs and are accessible to investigators via informal consent of the PIs. Data will be stored for at least five years after publication. Data are identified with manuscript ID, data generated, and name of the PI.

## Supplementary Materials

The following supporting information can be downloaded at: https://www.mdpi.com/article/10.3390/pharmaceutics17101337/s1, Figure S1: (A) ^1^HNMR in CDCl_3_ of PEG_44_–PPS_20_ block copolymer performed on a Bruker AVANCE (400 MHz) platform with Topspin software: δ = 1.35–1.45 (d, CH3 in PPS chain), 2.6–2.7 (m, -CH in PPS chain), 2.85–3.0 (m, -CH2 in PPS chain), 3.38 (s, -OCH3), 3.52–3.58 (t, -OCH2CH2S), 3.5–3.7 ppm (s, broad, -OCH2CH2 in PEG chain protons), 7.8–7.83 (m, 1H, pyridine group). (B) GPC trace in DMF of a PEG_44_–PPS_20_ block-copolymer synthesized for this work. The calculated PDI is reported on the graph and is = 1.09 for this batch of polymer. Figure S2: In vivo biodistribution of nMIC-DiD in the skin graft site of the mice further added in the study. The figure shows the whole-body imaging of an untreated mouse bearing a skin graft and 4 mice bearing a skin graft and treated with nMIC-DiD at 24 h (A) and 96 h (B) after administration (IP injection, 50 µL). Figure S3: Gallery of additional images for the skin grafts at POD 10 in treatment groups as indicated.

## Abbreviations

The following abbreviations are used in this manuscript:

^1^H NMR	Proton nuclear magnetic resonance
CoSE	Cosolvent evaporation
Dexa	Dexamethasone
DexP	Dexamethasone palmitate
DexSP	Dexamethasone sodium phosphate
DIANAs	Drug-Integrating Amphiphilic Nano-Assemblies
DLS	Dynamic light scattering
IVIS	In Vivo Imaging System
LISAI	Local Immune Suppression and Anti-inflammatory
MeOH	Methanol
nMICs	Nanomicelles
PEG	Poly(ethylene glycol)
PK	Pharmacokinetic
POD	Post-operation day
PPS	Poly(propylene sulfide)
RP-HPLC	Reverse-phase high-performance liquid chromatography
T1D	Type 1 Diabetes
