# Supplementary material for: Drug-Integrating Amphiphilic Nano-Assemblies: 3. PEG-PPS/Palmitate Nanomicelles for Sustained and Localized Delivery of Dexamethasone in Cell and Tissue Transplantations
Source: Pharmaceutics. 2025 Oct 16;17(10):1337. doi: 10.3390/pharmaceutics17101337 (PMC12567478; doi:10.3390/pharmaceutics17101337)
Supplement: Supplementary file 1 [file pharmaceutics-17-01337-s001.zip › pharmaceutics-3860151-supplementary.pdf]

Article

Drug-Integrating Amphiphilic Nano-Assemblies: 3. PEG-PPS/Palmitate Nanomicelles for Sustained and Localized Delivery of Dexamethasone in Cell and Tissue Transplantations

*G. Palummieri[1], S. Saadat[1], ST. Chuang[1], P. Buchwald[1,2] and D. Velluto[1]**

Supporting Information

A

B

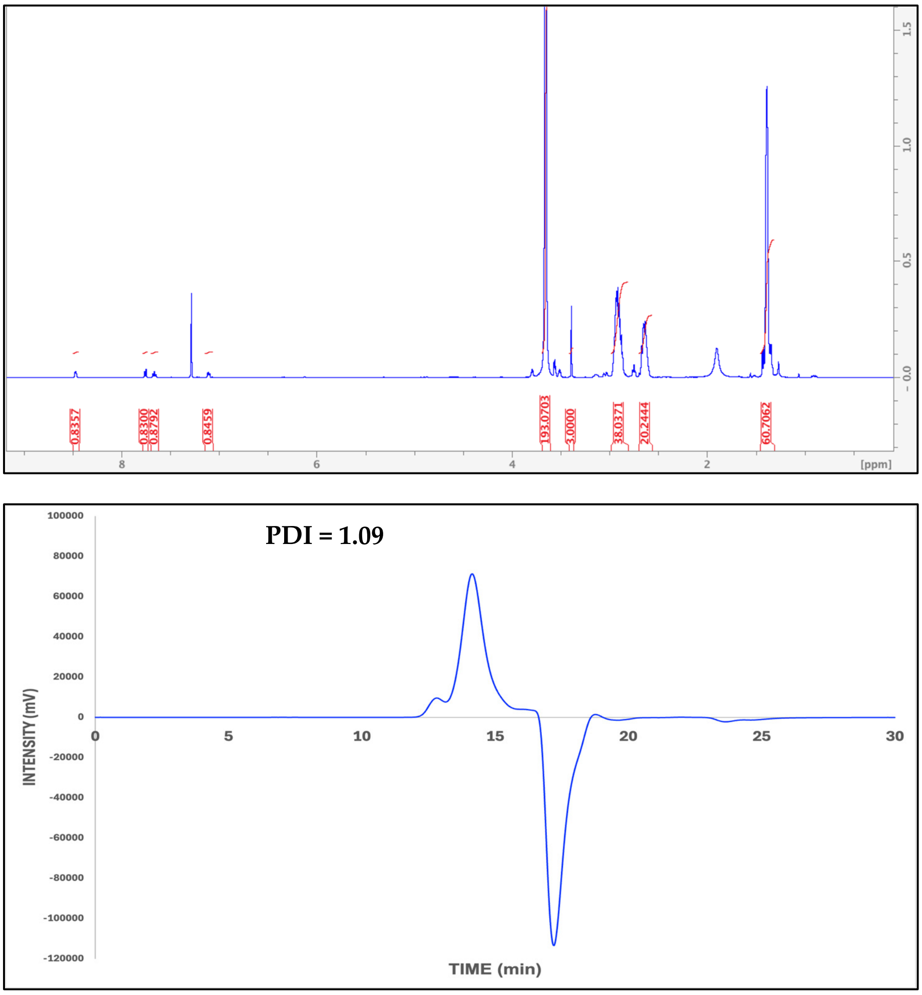

Figure S1: (A) ^{1}HNMR in $CDCl_3$ of $PEG_{44}PPS_{20}$ block copolymer performed on a Bruker AVANCE (400 MHz) platform with Topspin software: δ = 1.35–1.45 (d, CH3 in PPS chain), 2.6–2.7 (m, -CH in PPS

chain), 2.85–3.0 (m, -CH2 in PPS chain), 3.38 (s, -OCH3), 3.52–3.58 (t, -OCH2CH2S), 3.5–3.7 ppm (s, broad, -OCH2CH2 in PEG chain protons), 7.8–7.83 (m, 1H, pyridine group). **(B)** GPC trace in DMF of a $PEG_{44}PPS_{20}$ block-copolymer synthesized for this work. The calculated PDI is reported on the graph and is = 1.09 for this batch of polymer.

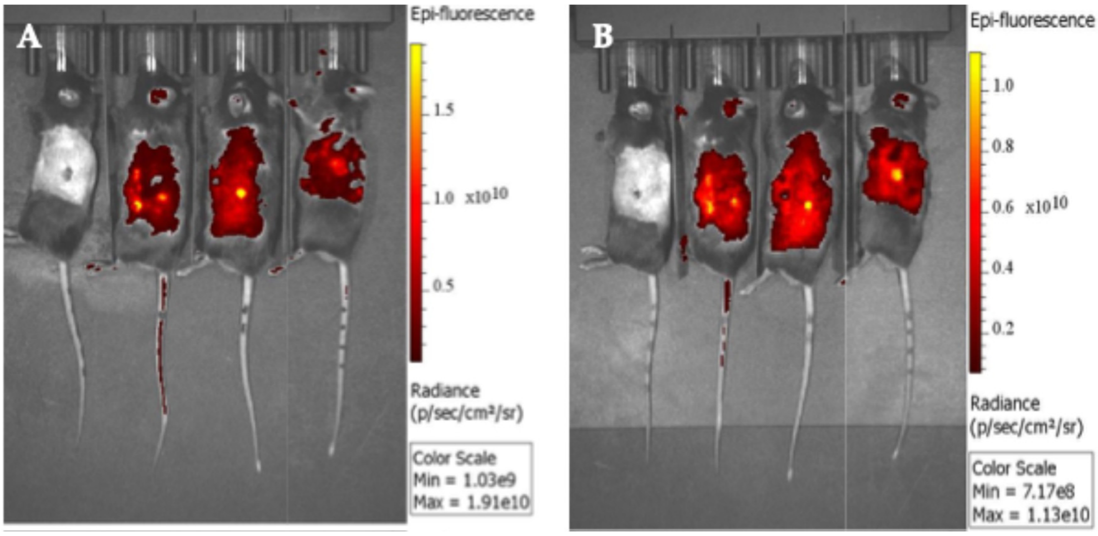

Figure S2: *In vivo* biodistribution of nMIC-DiD in the skin graft site of the mice further added in the study. The figure shows the whole-body imaging of an untreated mouse bearing a skin graft and 4 mice bearing a skin graft and treated with nMIC-DiD (mouse 2) at 24 h (**A**) and 96 h (**B**) after administration (IP injection, 50 mL).

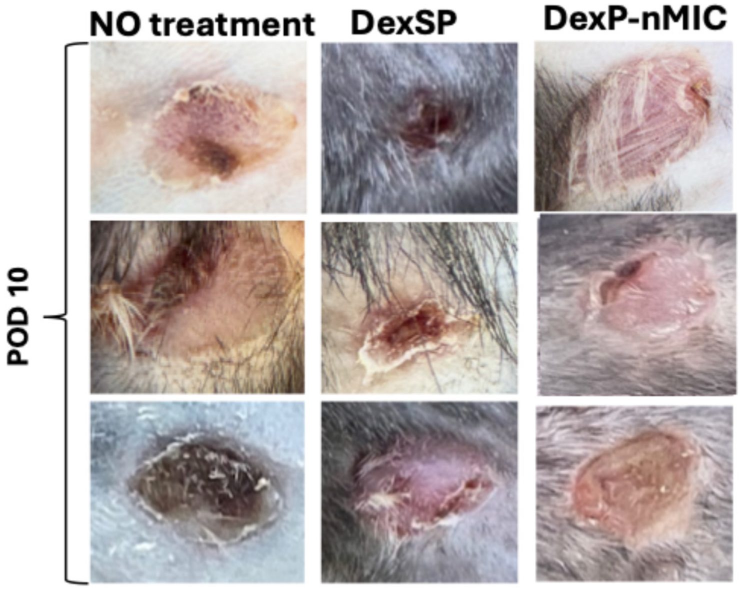

Figure S3: Gallery of additional images for the skin grafts at POD 10 in treatment groups as indicated.
